# Editorial: Trends in biomarkers for neurodegenerative diseases: Current research and future perspectives

**DOI:** 10.3389/fnagi.2023.1153932

**Published:** 2023-02-16

**Authors:** Suman Dutta, Miriam Sklerov, Charlotte E. Teunissen, Gal Bitan

**Affiliations:** ^1^International Institute of Innovation and Technology, Kolkata, India; ^2^Department of Neurology, University of North Carolina School of Medicine, Chapel Hill, NC, United States; ^3^Department of Clinical Chemistry, Vrije Universiteit Amsterdam, Amsterdam UMC, Amsterdam, Netherlands; ^4^Department of Neurology, David Geffen School of Medicine, Brain Research Institute, Molecular Biology Institute, University of California, Los Angeles, Los Angeles, CA, United States

**Keywords:** Parkinson's disease, Alzheimer's disease, cerebrospinal fluid (CSF), brain imaging (CT and MRI), Huntington's disease, amyotrophic lateral sclerosis (ALS), microRNA (miRNA), central nervous system (CNS)

Neurodegenerative diseases are characterized by a progressive decline in brain function and are a growing global threat (Jellinger, 2010). Early and accurate diagnosis of these conditions is vital for the development of therapeutic interventions to prevent disease progression and improve patient outcomes. However, many currently available biomarkers for these conditions have limited sensitivity and specificity, and are not clinically applicable during early disease stages (Hornung et al., 2020; Hansson, 2021).

A challenge for the diagnosis and treatment of neurodegenerative diseases is the difficulty accessing the brain of living individuals. For most of these diseases, analysis of brain tissue postmortem is required for accurate diagnosis. Major advancements have been made in the development of cerebrospinal fluid (CSF), imaging, and blood-based biomarkers for several neurodegenerative diseases (Ashton et al., 2020; Kaipainen et al., 2020; Dutta et al., 2021; Taha et al., 2022), which are proof of concept for the possibilities of early diagnosis. Moreover, advances in proteomics, transcriptomics, and metabolomics have provided valuable insights into the mechanisms of these conditions and have opened the door to the development of novel diagnostic and therapeutic approaches (Peplow and Martinez, 2021).

Collaborative efforts across the globe have helped elucidate the underlying mechanistic pathways of neurodegenerative diseases, yet many remain poorly understood (Figure 1). This Research Topic is a collection of research articles and reviews from diverse groups around the globe discussing recent developments and insights in the field of biomarkers for neurodegenerative diseases, their utility and limitations, and future directions toward implementation of advanced biomarkers in regular clinical practice.

**Figure 1:**
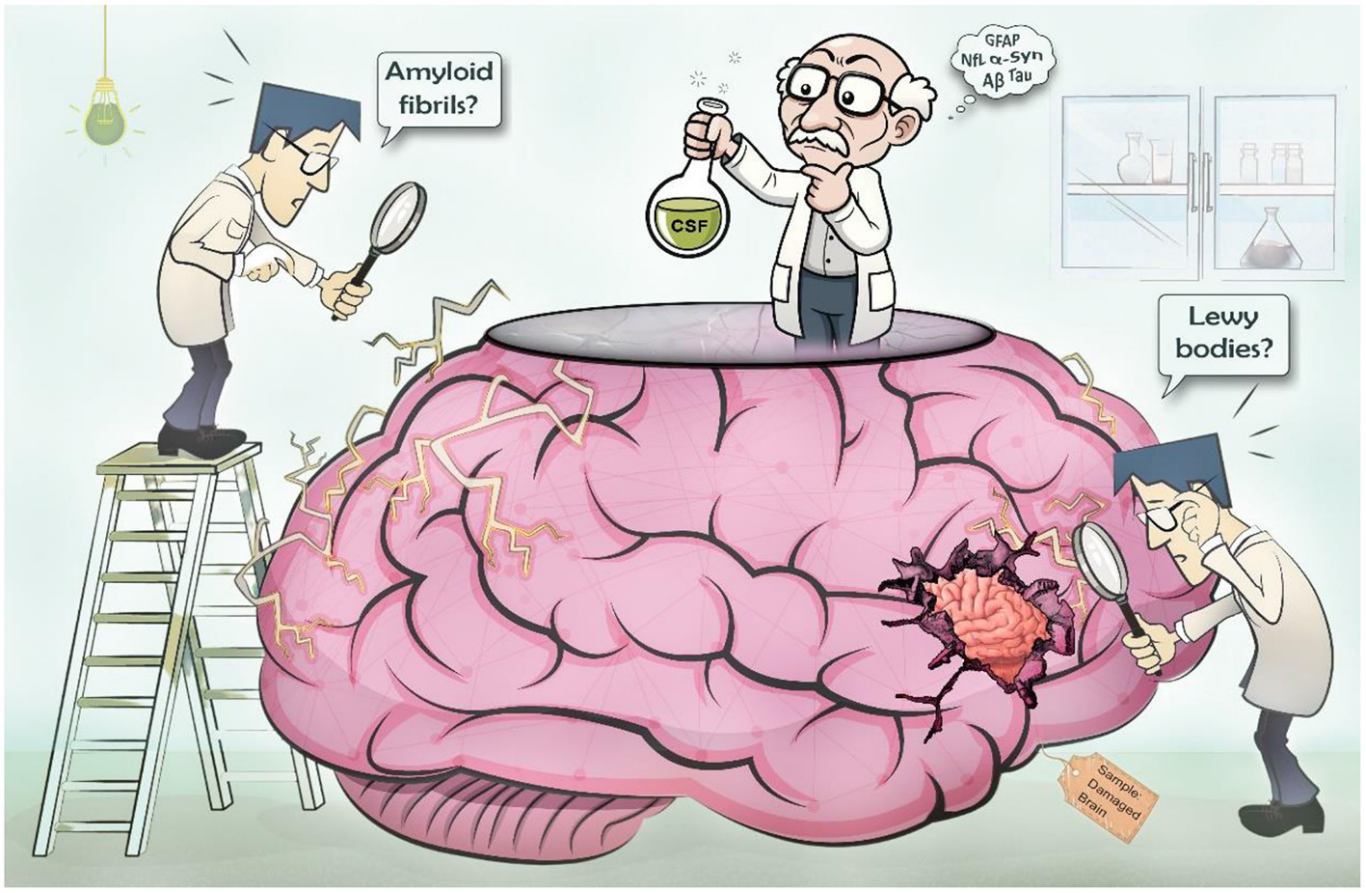
Challenges in identifying biomarkers for neurodegenerative diseases. Identifying biomarkers for neurodegenerative diseases is challenging because of the inaccessibility of the brain. As a result, there is a lack of detailed understanding of the underlying pathology. Additional challenges are variability in clinical presentation and overlapping symptoms, and the long latent period of many of these diseases making it difficult to pinpoint specific biomarkers for diagnosis and prediction of disease course.

Zhang, Ghose, et al. at Oxford University used deep-learning neural networks to identify blood proteins that could predict the now commonly used biomarker paradigm of amyloid, tau, and neurodegeneration (AT[N]) pathologies in AD. The researchers determined the AT[N] status in the brain and compared it to the corresponding blood biomarkers. The study found enrichment of proteins in five AD-associated clusters that could serve as surrogate blood biomarkers for AD. Along similar lines, to study the relationship between blood-based biomarkers of AD and cognition in motoric cognitive risk (MCR) syndrome, Chen et al. at MacKay Memorial Hospital, Taiwan, determined the levels of plasma Aβ42 and total tau. They found that plasma tau levels were significantly higher in the MCR and AD groups compared to the normal cognition group. These findings suggest that MCR and AD may share underlying pathology and cognitive function may be related to tau levels in MCR. A study by Parvizi et al. at the Medical University of Vienna, Austria, examined the potential of blood neurofilament light chain (NfL) and glial fibrillary acidic protein (GFAP), in detecting early neuropathological changes in AD. A panel combining plasma NfL and GFAP with known AD risk factors had a promising discriminatory power in distinguishing AD from healthy controls and predicting amyloid positivity.

A retrospective, case-selective clinical study by Li et al. at Tianjin Huanhu Hospital, China, aimed to differentiate AD from frontotemporal lobar degeneration (FTLD) using clinical, neuropsychological, and neuroimaging features. AD patients had a higher prevalence of vascular disease-associated factors and a higher percentage of Apolipoprotein E4 carriers. The findings suggest that dynamic evaluation of cognitive function, behavioral symptoms, and multimodal neuroimaging may help differentiate between AD and FTLD.

In a systematic review of preclinical *in vitro* and *in vivo* studies, Fathi et al. at Shahid Beheshti University of Medical Sciences, Iran, evaluated genes and mechanisms of neuroinflammation in Parkinson's disease (PD). The review identified several neuroinflammatory factors and molecular mechanisms contributing to the initiation and progression of PD, and potential therapeutic approaches against them. A systematic review focusing on different aspects by Asadi et al. at Tabriz University of Medical Sciences, Iran, attempted to identify validated competing endogenous RNA (ceRNA) loops in PD. The reviewed studies indicate that ceRNA axes have a significant impact on PD development and may be useful for the diagnosis and treatment of PD. Bioinformatic analysis of genes targeted in ceRNA axes showed that they were involved in processes such as the cellular response to metal ions, oxidative stress, and regulation of macromolecule metabolism. Heng et al. at the Second Affiliated Hospital of Soochow University, China, explored the association between osteoarthritis and PD through genetic characterization and functional enrichment. Using bioinformatics methods and datasets from the Gene Expression Omnibus database, they identified 71 common genes affecting both diseases, which were enriched in antigen processing and presentation, mitochondrial translation, the mRNA surveillance pathway, and nucleocytoplasmic transport. The study suggested that the gene WDR43 may be useful for the diagnosis of osteoarthritis and PD and that several immune cell types may be associated with the pathogenesis of both diseases. Zheng et al. at Beijing Tiantan Hospital, China, investigated whether abnormal α-synuclein (α-syn) deposition occurs in the oral mucosa of patients with multiple system atrophy (MSA) and whether α-syn and pathological forms thereof in the oral mucosa could be potential biomarkers for MSA. They found elevated levels of all tested α-syn species in patients with MSA compared to controls. Interestingly, the α-syn levels correlated negatively with disease duration, suggesting that they will be most useful at early disease stages.

The ongoing COVID-19 pandemic has been linked to a range of neurological complications, including cognitive impairment and neurodegenerative changes (Li C. et al., 2022). A review article by Silva et al. at Benemérita Universidad Autónoma de Puebla, Mexico, discusses the possibility of SARS-CoV-2 entering the central nervous system (CNS) through various neuroinvasive pathways, including the transcribrial route, the ocular surface, and the hematogenous system leading to the development of neurodegenerative diseases. The authors highlighted that the virus may also cause cytokine storms, neuroinflammation, and oxidative stress, which may increase the risk of developing diseases, such as AD and PD. Alvarez et al. at Marshall University, West Virginia, reviewed the diverse neurodegenerative changes associated with COVID-19 and highlighted the importance of major circulating biomarkers, associated with disease progression and severity. Their literature survey indicates that important CNS proteins, such as GFAP, NfL, and pT181-tau, and various inflammatory cytokines are altered significantly in COVID-19 patients. The review summarizes the current understanding of the neuropathological changes associated with COVID-19 and the potential use of biomarkers in identifying patients at risk for developing severe forms of the disease.

Huntington's disease (HD) is a genetic neurodegenerative disorder that is influenced by epigenetic changes, such as non-coding RNA expression and accelerated DNA methylation age (Bassi et al., 2017). Ghafouri-Fard et al. at Shahid Beheshti University of Medical Sciences, Iran, review the potential interactions among these different layers of the epigenome in relation to HD onset and progression. The authors discuss recent findings of micro RNA (miRNA) and long noncoding RNA dysregulation, as well as methylation changes and epigenetic age in HD.

In a study focusing on amyotrophic lateral sclerosis (ALS), Behler et al. at Ulm University, Germany, investigated the use of diffusion tensor imaging (DTI) as a progression biomarker. The study used Monte Carlo simulations to estimate the statistical power and sample size needed for DTI studies in ALS considering factors such as the number of scans per session, time intervals between measurements, and measurement uncertainties. The results showed that multiple scans per session can increase the statistical power of DTI studies in ALS, particularly in cases of high measurement uncertainty and small sample sizes. Addressing the question of developing biomarkers for the spectrum of diseases caused by tauopathy or TPD-43 proteinopathy, Wong et al. at the University of Toronto, Canada, used spectral-domain optical coherence tomography (SD-OCT) to examine the peripapillary retinal nerve fiber layer (pRNFL) thickness and macular retinal thickness in the eyes of participants with ALS, progressive supranuclear palsy (a tauopathy), and the semantic variant of primary progressive aphasia presumed to be caused by TDP-43 proteinopathy. Their data indicated that the TDP-43 group had a significantly thinner pRNFL in the temporal sector compared to the tauopathy group. miRNAs, a class of small non-coding RNAs, often are altered in neurodegenerative diseases and have the potential to be used as biomarkers (Li S. et al., 2022). Alvia et al. at Boston University School of Medicine compared the levels of 47 miRNAs in the prefrontal cortex of brain donors with chronic traumatic encephalopathy (CTE), ALS, both CTE and ALS, and control subjects. They found that 60% of the studied miRNAs were significantly different between the pathology groups, of which 75% were upregulated in both CTE and ALS. The identified miRNAs were involved in pathways related to inflammation, apoptosis, and cell growth/differentiation. Importantly, the largest change was in miR-10b, which was increased in ALS but not in CTE or CTE + ALS, suggesting that it could be used as a diagnostic biomarker.

Zhang, Chen, et al. at the Second Hospital of Hebei Medical University, China, studied the role of endothelin-1 (ET-1) in ALS and found that ET-1 and its receptors were expressed in the spinal cord of a transgenic mouse model of ALS and their expression changed as the disease progressed. In addition, ET-1 had a toxic effect on motor neurons in a cell model of ALS, which was rescued by selective ET-A or ET-B receptor antagonists. They also found that Cdkn1b (P27) and Eif4ebp1 could be used as biomarkers for understanding and identifying the pathogenesis of ALS responding to ET-1 intervention.

NfLs are proteins found in neurons and their levels have been shown to be useful in the diagnosis, prognosis, and monitoring of treatment response for a variety of neurological conditions (Gaetani et al., 2019). In a review article, Delaby et al. at Université de Montpellier, France, discussed the potential value of NfL assays in the diagnosis and management of patients with ALS, PD, frontotemporal dementia, and other neurologic diseases. The authors also described the added value of NfL compared to other biomarkers and proposed specific indications where NfL may be helpful in diagnostic and prognostic clinical decision-making. The authors pointed out the importance of establishing reference ranges for NfL levels in different biological samples, depending on factors such as age, body mass index, and the specific medical indication considered.

A study by Fernandez-Alvarez et al. at Pablo de Olavide University, Spain, examined the relationship between plasma markers of amyloid and neurodegeneration, intracortical myelin content, and resting-state functional connectivity in cognitively normal older adults. The researchers found that lower plasma Aβ42 and higher plasma NfL were associated with lower myelin content in certain brain regions. They also found that higher NfL levels were associated with altered functional connectivity between the insula and medial orbitofrontal cortex. The findings suggest potential links among plasma Aβ42 and NfL, intracortical myelin content, and functional connectivity in brain regions vulnerable to aging and neurodegeneration.

Overall, this Research Topic highlights recent development and innovation in the field of biomarkers for neurodegenerative diseases, including AD, PD, HD, ALS, and others. The Research Topic covers a range of biomarkers, including those that can be measured in the periphery, such as blood and spinal fluid. The Research Topic also covers the potential use of these biomarkers in clinical practice, including their potential to aid in the diagnosis, prognosis, and monitoring of disease progression and treatment response. Our goal as editors was to provide a comprehensive overview of the current state of the field and identify future directions for research and development in this important area and we hope the readers will find interest in the included articles and reviews.

## Author contributions

SD wrote the article. GB, MS, and CT reviewed the article and provided critical feedback. All authors contributed to the article and approved the submitted version.
